# Costs and effects of interventions targeting frequent presenters to the emergency department: a systematic and narrative review

**DOI:** 10.1186/s12873-019-0296-4

**Published:** 2019-12-30

**Authors:** Viola Korczak, Janani Shanthosh, Stephen Jan, Michael Dinh, Thomas Lung

**Affiliations:** 1 The George Institute for Global Health, UNSW Sydney, Level 5/1 King St Newtown NSW, Sydney, 2042 Australia; 2 Emergency Department, Royal Prince Alfred Hospital, Sydney, Australia; 3 Faculty of Medicine and Health, University of Sydney, Sydney, NSW Australia; 4 The Australian Human Rights Institute, UNSW Sydney, Sydney, Australia

**Keywords:** Emergency medicine, Frequent presenter, Health care costs, Health economics

## Abstract

**Background:**

Previous systematic reviews have examined the effectiveness of interventions for frequent presenters to the Emergency Department (ED) but not the costs and cost-effectiveness of such interventions.

**Method:**

A systematic literature review was conducted which screened the following databases: Pubmed, Medline, Embase, Cochrane and Econlit. An inclusion and exclusion criteria were developed following PRISMA guidelines. A narrative review methodology was adopted due to the heterogeneity of the reporting of the costs across the studies.

**Results:**

One thousand three hundred eighty-nine papers were found and 16 were included in the review. All of the interventions were variations of a case management approach. Apart from one study which had mixed results, all of the papers reported a decrease in ED use and costs. There were no cost effectiveness studies.

**Conclusion:**

The majority of interventions for frequent presenters to the ED were found to decrease ED use and cost. Future research should be undertaken to examine the cost effectiveness of these interventions.

## Background

Patients who frequently present to the Emergency Department (ED) represent a population for whom additional and specific programs could potentially yield significant potential economic and health benefits. However there is currently no agreed definition of ‘frequent presenter’ and definitions vary widely [1, 2] from three [3, 4] to more than eight [5] or ten [6, 7] presentations per year. Although frequent presenters to the ED have been found to be largely heterogeneous [8] [9], some generalisations can be made about this cohort. They are usually individuals who are from lower socio economic backgrounds [10] and are more likely to present with drug and alcohol [11], mental health [12] and chronic illnesses [10, 13, 14].

Despite the need for services to address this cohort, there is limited evidence of effective interventions targeted at this group. Some that have been used in this setting include case management [15], cognitive behaviour therapy (CBT) [16], or social worker involvement [17]. Limited research has been conducted on evaluating the costs and cost-effectiveness of these interventions compared to other hospital interventions.

This is an important topic for several reasons. First, health care spending is increasing disproportionately. Spending on health in Australia has grown by about 50% in real terms over the past decade compared with population growth of about 17% over the same period [18]. Emergency department use is on the rise and the causes are multifactorial, and include among other factors an ageing population and increase in chronic conditions [19]. Between 2012/2013 and 2016/2017 presentations to emergency departments in Australia increased by 3.7% on average each year [20]. Similar increases in emergency department use are evident in the USA [21]. Second, while frequent presenters may constitute a small percentage of the total of ED users they are often the most disadvantaged members of society [1]. In Australia for example, almost 24% of ED presentations are for patients living in the lowest SES groups [22].

The social determinants of health including income, education, employment and housing play a role in understanding this disadvantage, which is especially pertinent in the ED setting**.** One Canadian study found that homeless individuals have “greater than eight times the incidence of ED visits than their age and sex matched non homeless counterparts” [23]**.** Furthermore 50% of acute care is administered to the uninsured, who are usually the most economically disadvantaged [24]. The social determinants of health including housing are relevant to the provision of emergency department care. From an economic perspective it is timely to review which programs are benefiting this vulnerable group in the hope that they may be rolled out to maximise positive health and social outcomes.

The main aim of this research is to review the economic evaluations of interventions aimed at frequent presenters and to assess their cost implications and cost-effectiveness.

## Methods

A systematic review of the literature was conducted to assess interventions for frequent presenters to the ED and the associated costs of the intervention programs outlined. PRISMA guidelines were used for this systematic review [25].

There were two inclusion criteria. First, interventions had to be aimed at ‘frequent presenters’ to the Emergency Department. The numerical definition of ‘frequent presenter’ as specifically defined in each paper was used. Second, the cost of the intervention had to be assessed. Papers were included from all countries and languages, in adult populations over 18 years of age and if the study included more than 10 subjects.

Studies were excluded if the intervention was not based in the ED, did not focus on frequent presenters or did not include any data on the cost of the intervention. Review and opinion pieces were excluded as were papers set in paediatric populations or studies that included fewer than 10 patients. “Systematic reviews were excluded from the literature search to ensure that this review did not ‘double count’ individual papers. Another reason is because even though some literature reviews did have cost data, it was often summarised in one paragraph and it was not possible to assess the costing data adequately”. However we reviewed the bibliographies of literature reviews to ensure that relevant papers were not omitted.

A search was conducted in Pubmed, Medline, Embase, Cochrane and Econlit between June and December 2018. Grey literature was also reviewed and the reference lists of all relevant papers were screened. The following search terms was used in all databases: “(cost analysis OR cost effectiveness OR economic evaluation OR cost impact OR cost-benefit OR cost-utility) AND frequent* AND emergency”. The only limit on the search was ‘human’. There was no start date to the search and the end date was December 2018.

Two reviewers (VK and JS) independently reviewed the literature. Any discrepancies were resolved by a third independent reviewer (TL). An initial search of titles and then abstracts was carried out. Once abstracts were identified, the full texts of the papers were obtained and the final list for inclusion was decided. Data were independently extracted by the two reviewers (VK and JS) into a form in Excel developed prior to the review. Information on the year, country, setting, numerical definition of frequent presenter, interventions, comparator used in the study, healthcare system perspective, costs included, type of economic evaluation and outcome were included in the table. The reviewers met to verify that the extraction sheet was consistent. Any discrepancies in data extraction were resolved between the two reviewers. The reviewers (VK) and (JS) also assessed the papers using The Evidence Project risk of bias tool [26]. There was consensus between the authors on the quality of the papers and all the papers were included in the final analysis. The risk of bias table is included in the Additional file 1.

Once the table was completed it became evident that it was not possible to directly compare the outcomes of the studies due to the heterogeneity of reporting of the costs of the programs. A narrative review [27] was adopted and further tables were developed to synthesise the information on this topic.

## Results

**Fig. 1:**
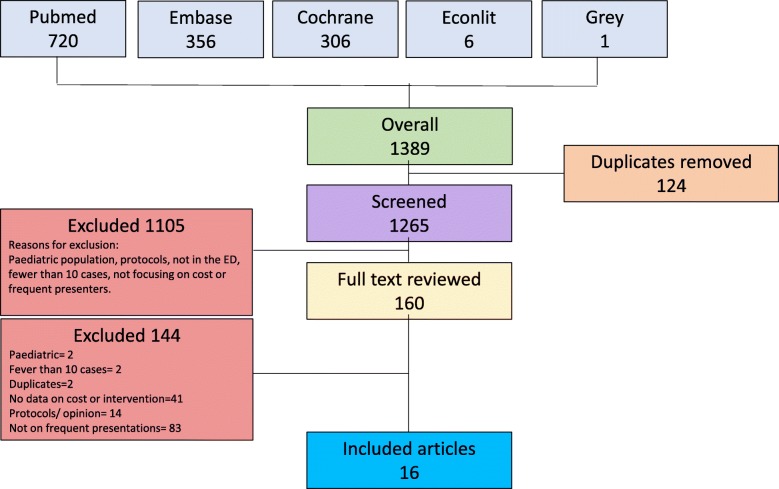
PRISMA diagram of results

The search term yielded 1389 papers in total. Once the titles were screened, 160 abstracts were reviewed and 16 papers were included in the review. Figure 1 includes the PRISMA flowchart of the search results. Table 1 summarises the main outcomes from the literature review. Of the 16 papers included in the review, all the papers were in English. All of the studies were from the US except two from Sweden [28, 29] and one from Australia [30].

**Table 1:** Study characteristics

Study (Yr)	Country	Setting	Number of subjects in the study	Intervention period	Comparator
Crane (2012)	USA	200 bed not for profit hospital.	36 (from initial group of 255)	July 2009–June 2010	Pre and post
DeHaven (2012)	USA	1 ED in Dallas	574 (265 intervention and 309 controls)	April 2003–July 2004	Controls
Edgren (2016)	Sweden	5 counties in Sweden.	Group One used Zelen’s design and had 7280 in intervention group and 3508 in control group. Group two used RCT design and had 934 in intervention group and 459 in control group.	2010- March2014.	Controls
Enard (2013)	USA	Nine Memorial Hermann EDs in the Houston area	13,642 participants (1905 in intervention and 11,373 in control group).	Nov 2008- April 2011	Pre and post
Grimmer-Somers (2010)	Australia	One metropolitan health region	37 patients	18 months from 2007 to 2009	Pre and post
Grover (2018)	USA	ED community hospital with 225 bed hospital in suburban area.	158 in intervention.	Oct 2013- June 2015	Pre and post.
Hardin (2017)	USA	Inner city tertiary hospital. 80,000 annual ED visits.	339 in intervention	Nov 2012-Dec 2015	Pre and post
Lin (2017)	USA	Brigham and Women’s hospital. Large urban acaedemic medical centre	72 (36 in intervention and 36 in control)	Oct 2014-April 2015	Controls.
Murphy (2013)	USA	Regional medical and trauma centre. 644 beds and 80,000 ED visits per yr.	141 (76 extreme and 65 frequent ED users)	Jan 2008- Dec 2010	Pre and post.
Navratil-Strawn (2014)	USA	Patients enrolled in an insurance scheme.	14,140 (7070 participants and an equal number of controls)	June-Nov 2011	Controls.
Okin (2000)	USA	San Francisco General Hospital.	53 patients.	June 1995–June 1996	Pre and post .
Reinius (2012)	Sweden	Karolinska University Hospital with 90,000 visits per yr.	268 patients (211 in intervention and 57 in control group).	Sep 2010- Sep 2011	Controls
Seaberg (2017)	USA	Urban ED with 57,000 presentations per yr.	318 (184 in intervention and 134 controls).	Jan- June 2015	Controls.
Shumway (2008)	USA	San Francisco General Hospital. Sole Level 1 trauma hospital in the county.	252 (167 in intervention and 85 to usual care)	March 1997 to Feb 1999	Controls.
Stokes-Buzzelli (2010)	USA	Urban hospital with 95,000 ED presentations per yr.	36 patients	June 2005–July 2007	Pre and post
Tadros (2012)	USA	One urban hospital.	51 patients	Dec 2006-June 2009	Pre and post

The Evidence Project risk of bias tool [26] showed that the majority of the papers were cohort studies. Five of the studies randomly assigned participants. One study reported on follow up rates, in the other papers follow up was either not applicable because of study design or not reported. The table is included in the Additional file 1

### Study characteristics

The majority of the studies were conducted at a single site except for Edgren [29] which was carried out across five counties in Sweden and Enard [31] which was carried out in nine emergency departments. One other study [3] used patients from an insurance database to assess costs. The majority of the studies included in the analysis are from the US. The findings from the US studies may have different implications from the Swedish and Australian studies as the US does not have a universal healthcare system. Therefore the cost savings identified in these studies could be for both health care services provided as well as individual out of pocket costs, which would be significantly less in a universal health care system.

The studies ranged in size from 36 [32, 33] to 14,140 [3] participants in an insurance scheme. All the studies were carried out between 1995 and 2015 with the majority being carried out after 2005.

The interventions are described in the table but all were based on a case management approach. Seven of the studies had controls alongside the intervention group [3, 15, 28, 29, 34–37]. The majority of the studies were pre and post studies and were non-randomised. Only five [15, 28, 29, 35, 38] of the studies were randomised and one of those was a pilot study [35].

### Definitions

As seen previously [39] the definition of frequent presenter to the ED varied markedly. All the papers targeted ‘frequent presenters’ generally, rather than focusing on subgroups of frequent presenters such as the homeless or psychiatric services [40]. Table 2 provides a summary of the definitions used in the papers. These varied from 2 presentations per year [34] to more than 10 presentations per year [41]. Some papers simply identified patients “with the greatest number of visits” [33] or “who were known to use services in an unplanned manner” [30] but did not quantify the definition.

**Table 2:** Frequent presenter definitions

Author	Year	Frequent Presenter’ Definition
Crane	2012	≥6 visits/ year
DeHaven	2012	≥2 visits/ year
Edgren	2016	≥3 visits in previous 6 months
Enard	2013	Extracted data from > 5 visits pre intervention period.
Grimmer-Somers	2010	Individuals known to use public hospital ED services in an unplanned manner.
Grover	2018	≥10 visits in 12 months, ≥6 visits in 6 months, ≥4 visits in 1 month or concerned ED use.
Hardin	2017	≥3visit/ year
Lin	2017	Patients with the most ED visits in the previous month and previous year.
Murphy	2013	Frequent = 3–11 visits per year, Extreme = ≥12 visits/year preceding year of enrolment.
Navratil-Strawn	2014	≥3 visits/ year in the previous 12 months
Okin	2000	≥5 visits/ year
Reinius	2012	≥3 visits during 6 months before inclusion.
Seaberg	2017	≥5 visits/ year
Shumway	2008	≥5 visits/ year
Stokes-Buzzelli	2010	Patients with the most ED visits.
Tadros	2012	≥10 visits/ year

### Intervention outcomes

Table 3 summarises the interventions and whether each study led to a reduction in ED use or cost. All of the included studies used a variant of a case management approach. Different names were used such as care coordination or acute care plans. The details varied from study to study but often included sessions with a social worker or nurse with referrals to specialist services and telephone follow up. Some studies included additional services such as mentoring and goal setting [30] or assistance with housing [15, 41, 42]. The majority of the studies yielded findings of decreased ED use and costs. However three of the studies found either mixed results [29], a decrease in ED use but not inpatient use [15] or a decrease in ED use but not in outpatient use. No ‘adverse events’, such as costs being diverted to other areas were examined.

**Table 3:** Summary of Interventions and Outcomes

Intervention	Crane (2012)	DeHaven (2012)	Edgren (2016)	Enard (2013)	Grimmer-Somers (2010)	Grover (2018)	Hardin (2017)	Lin (2017)	Murphy (2013)	Navratil-Strawn (2014)	Okin (2000)	Reinius (2012)	Seaberg (2017)	Shumway (2008)	Stokes-Buzzelli (2010)	Tadros (2012)
Case management/ care coordination/ acute care plans	X	X	X	X	X	X	X	X	X	X	X	X	X	X	X	X
Financial assistance (social security)											X					
Goal setting					X											
Group therapy	X										X			X		
Harm reduction services											X					
Housing											X			X		X
Individual visits / CHW/ Nurse	X	X		X				X			X	X		X		X
Mentoring					X											
Monetary assistance with pharmacy, lab tests and other medical costs		X														
Multidisciplinary						X	X	X	X					X	X	
Problem solving					X											
Referral to specialists and other services including social services		X	X	X				X	X	X	X	X	X	X		X
Telephone	X	X	X					X	X	X		X	X			X
Transportation					X											X
**Change in ED use**	**↓**	**↓**	**Mixed**	**↓**	**↓**	**↓**	**↓**	**↓**	**↓**	**↓**	**↓**	**↓**	**↓**	**↓ ED use but not in inpatient**	**↓**	**↓**
**Magnitude of change**	ED use dropped from a rate of 0.58 per patient per month to 0.23**	Fewer ED visits, 0.93 vs 1.44**	**12% decrease in hospitalisation (95%CI 4–19%)**	**V**	**30 ED presentations by 11 users in 2006 dropped to 22 presentations by 9 users in 2009.**	830 fewer ED visits and a 49.26% change**	Mean difference of 5.5 and a 37.4% change**	**35% fewer ED visits**	frequent users decreased by 5 visits (95% CI of 2–5); extreme users decreased by 15 visits (95% CI of 13–17)*	ED visits decreased by 178/1000 visits; hospital admission by 53/1000 visits*	Median number of visits decreased from 15 to 9 visits (95% CI of 3–7 visits)**	**RR 0.77 (95% CI of 0.69–0.86)**	13.2% decrease in ED use from 1148 to 996 visits**	**V**	Visits decreased by 25% from 67.4 to 50.5 (95% CI 0.3 to 33)*	Visits decreased by 28.1% from 199 to 143**
**Change in cost**	**↓**	**↓**	**Mixed**	**↓**	**↑ outpatient clinic use. ↓ crisis ED use.**	**↓**	**↓**	**↓**	**↓**	**↓**	**↓**	**↓**	**↓**	**Effective but not cost saving**	**↓**	**↓**
**Magnitude of change**	Hospital charges dropped from $1167 per patient per month to $230**	$1188 vs $446**	**V**	**V**	**NR**	$2,785,690 absolute change and 47.81% change**	Mean difference of $6290 and 47.9%**	**ED costs were 15% lower**	Decrease in frequent ED use by $1285 (95% CI of $492–$2364); extreme ED use decreased by $6091 (95% CI of $4298–$8998)*	**A saving of $21 per member per month for ED visits or $59 per member per month on admissions.**	ED costs decreased from $4124 to $2192 (95% CI $1013 to $2459 to); and inpatient costs decreased from $8330 to $2786 (95% CI $0 to $8330).**	45% decrease in total cost per patient**	26.6% decrease (95% CI 26.1 to 27%)**	**V**	decreased by 24% from $64,721 to $49,208 (95% CI $83 to -$30,943)*	**12.7% decrease in costs from $413,410 to $360,779**

*V* Variable rate

*NR* Not reported

*p < 0.05

**p < 0.01

Entries in boldface summarise the main outcomes

The magnitude of the change in costs and effects is captured at the bottom of Table 3. While there is heterogeneity in reporting, it was possible to summarise some of the degree of change across the studies as a result of the various interventions.

### Economic outcomes

Table 4 summarises the main economic outcomes. All of the included studies examined cost from the healthcare system perspective. Though one of the papers [15] included ‘cost effectiveness’ in the title, the paper did not report an incremental cost effectiveness ratio (ICER). Two of the studies [3, 42] reported on return on investment (ROI). All of the studies included some form of cost analysis without analysis of effectiveness.

**Table 4:** Economic outcomes

Study (Yr)	Country	Social Economic Background	Perspective	Cost variable included in analysis	Type of economic evaluation	Outcome
Crane (2012)	USA	Low income, uninsured.	Healthcare	Hospital charges ($1167 per month pre-intervention, $230 post-intervention); cost of program ($66 K)	Cost analysis	ED use dropped by 0.25 per patient per month 0.23 and hospital charges dropped from $1167 per patient per month to $230.
DeHaven (2012)	USA	Uninsured	Healthcare	Indirect costs (sum of costs for all ED visits for the year, includes fixed costs related to building maintenance, staffing and utilities)	Cost analysis	Intervention enrolees of the PAD program had significantly fewer ED visits (0.93 vs 1.44). Direct hospital costs around 60% less ($1188 vs 446). Indirect costs 50% less ($313 vs $692).
Edgren (2016)	Sweden	“Screening aimed to identify patients who seemed to be lacking in health literacy, sought care at an improper level, or from too many providers”.	Healthcare	Costs of conducting maintenance activities ($13,950.42), total program cost ($54,284.31). Per-client discretionary costs for transport, equipment, medications and interpreters ($250 per person).	Cost analysis	The traditional design showed an overall 12% decreased rate of hospitalization, which was mostly driven by effects in the last year.
Enard (2013)	USA	Publically insured (Medicaid), uninsured (self pay), or covered by a local public health benefit that subsidises medical costs for eligible residents.	Healthcare	Prior to enrolment: ED charges ($8,453,761), inpatient charges ($8,453,761). Post-intervention: ED charges ($3,041,473) and inpatient charges ($5,405,175).	Cost analysis	The savings associated with reduced PCR-ED visits were greater than the cost to implement the navigation program.
Grimmer-Somers (2010)	Australia	Unplanned ED use, crisis inpatient admission, poor attendance at primary health and/ or outpatient clinics, unmanaged chronic disease, medication misuse, vulnerable social circumstances.	Healthcare	Gross charges and expenses, ED service charges and expenses, IP service charges and expenses, outpatient service charges and expenses.	Cost analysis	Staff spent 34 h with each client, costing $1700 each. Crisis ED and inpatient admissions decreased. Planned outpatient clinic use increased.
Grover (2018)	USA	Patients who demonstrated a propensity for future problematic ED encounters such as violence in the ED or prescription forgery.	Healthcare	Average direct costs per patient for intervention and control groups.	Cost analysis	ED and hospital charges decreased by 5.8 million dollars (41% reduction)
Hardin (2017)	USA	Patients who would benefit from a Complex Care Map	Healthcare	Direct treatment costs (wages, salaries, materials); indirect costs (those incurred as part of the production process (e.g. admin costs, maintenance costs)	Cost analysis	ED mean visits decreased 43%, inpatient admission decreased 44%. Gross charges decreased 45%, direct expenses decreased 47%.
Lin (2017)	USA	NR	Healthcare	Hospital service costs	Cost analysis	Average ED direct costs 15% lower for intervention patients. Average inpatient costs per patient 8% lower.
Murphy (2013)	USA	NR	Healthcare and fire department	Health care system costs - total costs for transport or non-transport responses based on predicted or actual call volume.	Cost analysis	Frequent and extreme users decreased in ED visits (5 and 15 respectively) and direct treatment costs ($1285) leading to significant hospital cost savings.
Navratil-Strawn (2014)	USA	Insurance scheme	Healthcare	Hospital inpatient and outpatient Medicare costs (not charges). ED physician costs not included in this study.	Cost analysis and ROI	Participants had greater reduction in ED visits (*p* = 0.003) and hospital admissions (*p* = 0.002) and increased office visits (p = < 0.001). ROI of 1.24.
Okin (2000)	USA	Program aimed to decrease homelessness, decrease alscohol and substance use and improve linkages to primary care providers, reduce health care utilisation and enrol patients without meical insurance to medicaid.	Healthcare	Medical inpatient costs, psychiatric emergency costs, psychiatric inpatient costs, medical outpatient costs, physicians’ professional fee costs, non EDCM costs	Cost analysis and ROI	Median number of ED visits decreased from 15 to 9 (p < 0.1) and median inpatient costs decreased from $4330 to $2786 (*p* < 0.1). ROI of $1.44.
Reinius (2012)	Sweden	NR	Healthcare	Ambulance and hospital charges as proxy for cost of care. No evaluation of individual insurance status or reimbursements.	Cost analysis	Intervention reduced the total healthcare costs for per person hospital admissions by 45%.
Seaberg (2017)	USA	NR	Healthcare	Total healthcare cost, primary and secondary care visit costs for outpatient care	Cost analysis	ED visits decreased overall with a larger decrease in the intervention group (by 13.2%) compared to the control group (by 4.5%).
Shumway (2008)	USA	Subjects had psychosocial problems that could be addressed with case management (problems with housing, medical care, substance abuse, mental health disorders or financial entitlements).	Healthcare	Total costs of the intervention and total cost per person	Cost analysis	Reductions in ED use and cost did not translate to reductions in inpatient use, which represent a larger proportion of total hospital service use.
Stokes-Buzzelli (2010)	USA	89% of the study population had substance abuse issues.	Healthcare	ED charges	Cost analysis	ED charged decreased by 24% (from $64,721 to $49,208). The number of lab studies ordered decreased by 28%. The number of average ED visits decreased by 25%.
Tadros (2012)	USA	NR	Healthcare	Total healthcare costs for hospital admissions	Cost analysis	Pre-hospital based case management system is effective in decreasing transport by frequent presenters but had only a limited impact on use of hospital services.

*NR* Not reported

All of the papers in the review comment on the cost implications of the interventions and these are summarised in Table 4. It was unclear whether the costs reported in the studies incorporated the costs of the program. Crane with twice weekly meetings cost $66,000 for the year for an intervention for 36 patients [32]. Grimmer- Sommers entailed an equivalent amount for 37 patients $63,434 [30]. Lin reported 36 people in the intervention arm with an annualised cost of the program of $55,115 [35], though it is unclear how often the patients received the intervention. The program by Murphy cost $265,680 for the year [43] for a broad multidisciplinary program for 141 patients. Okin reported a total cost of $296,738 for 53 patients over one year for a comprehensive case management program. Navaratil-Strawn included the large dataset of insurance users and cost $2.75 million to implement [3].

Other papers reported a percentage change or decrease as a result of the program. Crane [32] reported hospital charges dropped from $1167 to $230 per patient per month. Dehaven [34] reported direct hospital costs decreased by 60% and indirect costs by 50%. Grover [44] reported a 41% reduction in hospital charges. Hardin [45] reported 45% decline in gross charges and 47% reduction in direct expenses. Lin [35] found a 15% reduction in ED direct costs as a result of the intervention. Reinus [28] reported a 45% decrease in per patient hospital costs. Seaberg [38] found a decrease in overall healthcare costs of 26% in the intervention group (though the control group also saw a reduction of 17.5%). Stokes- Buzzelli [33] reported a 24% decrease in ED charges and Tadros [41] a 32.1% decrease in charges.

Costs were reported in different ways which made it difficult to compare across studies. Some papers [32] reported a decrease in cost per patient per month as a result of the intervention. Dehaven [34] and Hardin [45] reported a decrease in direct (wages, salaries, materials) and indirect costs (administration costs, insurance and maintenance costs) as a result of the intervention. While Grover [44] reported a total decrease in programme costs which was due to a 49% reduction in visits attributable to the intervention. The cost saving came from the result of fewer patients presenting to the ED and needing investigations and admission. Others [3, 42] reported a return on investment (ROI) which were similar in both studies, $1.24 [3] and $1.44 [42]. This was measured by dividing the total program savings by the total program costs. Overall however, as there were multiple differences in settings and methodology, comparisons of findings across the studies need to be made with caution.

## Discussion

This review showed that interventions targeting frequent presenters in emergency departments can have an impact in saving health care costs. Of the 16 papers included in the review, all of them reported on costs but there is no data on cost effectiveness. Future studies should standardise the way that costing information is reported so that costs may be compared between interventions within the same healthcare system. For example reporting costs in individual units such as for staff, medications and investigations. The CHEERS [46] statement seeks to standardise the way in which economic evaluations are reported.

The study by Edgren [29] stands apart from the other interventions as it showed mixed results. Patients were recruited from five counties in Sweden and different study designs were used (Zelen’s and randomised controlled trial (RCT)) “depending on local requirements and preferences” [29]. The study arms were analysed and reported separately. There was no difference in the first two years but decreases in the number of days in hospital and average cost were found in the third year. However, overall there was “no significant difference in either total healthcare costs or the number of days in hospital” [29].

Despite using a mix of interventions across the studies, most of the approaches led to a decrease in ED presentations and costs. A case management approach which linked patient services was the standard model employed which generally included contact with a nurse or community health worker, referral to services and telephone follow up. Other approaches included financial assistance in the form of linkages to social security entitlements [42], financial reimbursement for medical (mainly laboratory and pharmacy) services [34], goal setting [30] and housing support [15, 41, 42].

The level of patient interaction varied across the studies. Crane included twice weekly appointments with a multidisciplinary team [32], while Dehaven [34] outlined monthly meetings with a community health worker. The intervention by Reinius [28] included weekly or biweekly contact with patients. While the study by Seaberg [38] followed patients up at 2 weeks and 12 months from initial contact. In Stokes-Buzzelli [33] patients were contacted at least monthly. The other studies in the review did not specifically mention the frequency of patient contact. The studies which incorporated more frequent contact with patients still showed a decrease in ED use and cost.

Six of the interventions included a multidisciplinary team [15, 33, 35, 43–45]. The remaining interventions were led by nurses, social workers or community care coordinators. Regardless of the way the intervention was carried out, it tended to decrease ED use. Furthermore a multidisciplinary approach while likely more expensive to carry out, apart from Shumway [15] still led to cost savings. This could be as a result of multidisciplinary committees being formed by individuals already employed by the hospital, thereby not increasing program costs as the staff are already employed. Usually this would be included as a cost as it represents an opportunity cost as these staff members could be doing something else with their time.

An advantage of these case management programs is that patients can be assessed away from a busy and often over crowded emergency department. This could potentially lead to better management and fewer repeat investigations, which would likely be contributing to increasing costs.

This study is different to others in that it draws together the evidence of costing interventions for frequent presentations to the ED. Some papers outlining interventions have included some information regarding cost or cost effectiveness but as far as we are aware there has been no previous systematic literature review summarising the cost implications of interventions for frequent presenters. Given the mounting budgetary pressures on health systems worldwide, these findings provide guidance for health care decision makers addressing the financial pressures exerted in the delivery of emergency room care. The second strength was that it was a systematic review following PRISMA guidelines which were rigorous.

There were however a few limitations of this study. First, most of the studies were carried out in the US. The findings from the US setting may not translate directly to other countries with a different health care system. The drivers for reducing frequent presentations to the ED in the US (a user pays largely privatised system) would be different to a single payer system like that of the UK or Australia. Second, the majority of the studies included a simple cost analysis rather than cost effectiveness studies and may overlook presentations to other health care settings including allied health and potential health outcomes resulting from reduced ED presentations. Third, fewer than half of the studies used controls. The majority of the studies were pre and post with the same cohort and therefore may not adequately account for secular trends or regression to the mean [13, 47]. Another limitation is the absence of a clear definition of ‘frequent presenter’. A definition of ‘frequent presenter’ is lacking in the literature. As with medicine, it is important to define the problem in order to find a solution, yet there is no consensus in the literature. This is something the authors are currently working on in other research they are undertaking and hope to develop an inclusive and definitive classification that can be widely used.

## Conclusion

The main conclusion to draw from this study are that all of the interventions have an element of case management, most of which were shown to be cost saving. A range of interventions based on case management approaches was adopted and no difference was found. The cost savings were reported in different ways across the studies, either in the number of ED presentations, direct or indirect hospital costs. Future research should focus on conducting cost-effectiveness analyses to aid decisions about whether an intervention should be offered to frequent presenters to the ED. Such research would also shed light on the clinical effectiveness of the different approaches taken and potentially provide an economic case for funding such programs. Despite the research in this area there is no definitive approach or program for this population group. A cost effectiveness study which assesses effectiveness alongside costs would do much to add to the current evidence on the best methods to assist this population group.

## Supplementary information

**Additional file 1:** Risk of Bias Assessment.

## Data Availability

The datasets used and/or analysed during the current study are available from the corresponding author on reasonable request.

## Abbreviations

CBT: Cognitive behaviour therapy
ED: Emergency department
ICER: Incremental cost effectiveness ratio
RCT: Randomised control trial
ROI: Return on investment
